# Imbalances in the oral health workforce: a Canadian population-based study

**DOI:** 10.1186/s12913-024-11677-7

**Published:** 2024-10-07

**Authors:** Neeru Gupta, Pablo Miah

**Affiliations:** 1 University of New Brunswick, Tilley Hall room 20, Fredericton, E3B 5A3 Canada; 2 New Brunswick Institute for Research, Data and Training (NB-IRDT), Keirstead Hall suite 304, Fredericton, E3A 5A3 Canada

**Keywords:** Health workforce, Oral health care providers, Dentists, Dental staff, Dental hygienists, Rurality index, Gender equity, Gender wage gap, Universal health coverage

## Abstract

**Background:**

In Canada, a new federal public dental insurance plan, being phased in over 2022–2025, may help enhance financial access to dental services. However, as in many other countries, evidence is limited on the supply and distribution of human resources for oral health (HROH) to meet increasing population needs. This national observational study aimed to quantify occupational, geographical, institutional, and gender imbalances in the Canadian dental workforce to help inform benchmarking of HROH capacity for improving service coverage.

**Methods:**

Sourcing microdata from the 2021 Canadian population census, we described workforce imbalances for three groups of postsecondary-qualified dental professionals: dentists, dental hygienists and therapists, and dental assistants. To assess geographic maldistribution relative to population, we linked the person-level census data to the geocoded Index of Remoteness for all inhabited communities. To assess gender-based inequities in the dental labour market, we performed Blinder-Oaxaca decompositions for examining differences in professional earnings of women and men.

**Results:**

The census data tallied 3.4 active dentists aged 25–54 per 10,000 population, supported by an allied workforce of 1.7 dental hygienists/therapists and 1.6 dental assistants for every dentist. All three professional groups were overrepresented in heavily urbanized communities compared with more rural and remote areas. Almost all dental service providers worked in ambulatory care settings, except for male dental assistants. The dentistry workforce was found to have achieved gender parity numerically, but women dentists still earned 21% less on average than men, adjusting for other characteristics. Despite women representing 97% of dental hygienists/therapists, they earned 26% less on average than men, a significant difference that was largely unexplained in the decomposition analysis.

**Conclusions:**

Accelerating universal coverage of oral healthcare services is increasingly advocated as an integral, but often neglected, component toward achieving the health-related Sustainable Development Goals. In the Canadian context of universal coverage for medical (but not dentistry) services, the oral health workforce was found to be demarcated by considerable geographic and gendered imbalances. More cross-nationally comparable research is needed to inform innovative approaches for equity-oriented HROH planning and financing, often critically overlooked in public policy for health systems strengthening.

## Background

Achieving universal coverage of essential healthcare services is a centrepiece of the Sustainable Development Goals (SDGs) global health agenda (Target 3.8) [1–3]. A number of tracer indicators have been developed and adopted internationally over several years to summarize inequalities and monitor progress toward universal coverage across a range of health domains [1]. These include tracers of service access, coverage, and financial risk protection for selected health conditions such as maternal and newborn care, HIV and tuberculosis treatment, and cancer detection — and disaggregated by equity dimensions including gender and place of residence (typically rural versus urban regions). They also include tracers of service capacity, counting per capita densities of hospital beds and of healthcare workers, disaggregated by place of residence. Given data limitations in many countries, the focus for monitoring healthcare worker density and distribution has tended to be constrained to physicians [1]. None of the official SDG targets to date are specific to oral health needs or service providers, although global monitoring efforts are gradually expanding to distinguish dentistry personnel (Indicator 3.c.1) [4]. It is increasingly advocated that access to oral health services must be an integral component of universal health coverage within and across populations [2, 5–7]. Policy and research attention for accelerating universal coverage of dentistry services has been limited, particularly in comparison with medical services [8, 9]. The first-ever global strategy on oral health, launched by the World Health Organization in 2022, identified the need for enhanced data and evidence to inform scaling up of universal dental coverage [10, 11]. The strategy furthered that, in many countries, inadequate attention has been paid to health workforce availability and accessibility, including dentists and other oral health service providers [10].

The global burden of oral diseases is high and shows few signs of improvement over time, yet remains a largely neglected global population health challenge [12, 13]. Oral ill-health inequities are observed across low- and middle-income countries, and also persist in high-income countries [14]. For example, in the United States, children in some minority racial groups continue to have higher rates of oral disease and unmet dental care needs [15]. A study in Norway reported significant social correlates of dental disease among older adults [16]. In Canada, poorer oral health outcomes have been found among persons with lower income and among those experiencing food insecurity — a disparity linked, in turn, to vulnerable persons being less likely to access preventive dental services (e.g., routine cleanings), seek treatment for initial periodontal conditions (e.g., pain, microbial infection), or receive early diagnosis for severe health outcomes (e.g., oral cancer) [8, 17–19]. Beyond financial risk protection, tracking progress toward universal dental coverage requires baseline evidence on human resources for oral health (HROH), starting with numbers and distributions of dentists and allied dental personnel (e.g., dental therapists, hygienists, and assistants) [20].

In their seminal paper, Zurn et al. differentiated four domains of health workforce imbalances as crucial to enhancing service access and equity: professional/skills mix, geographical, institutional, and gender [21]. In relation to HROH planning, despite the importance of optimizing skills mix, much of the existing research literature concentrates on dentists [20]. Improving the geographic distribution and demographic diversity of the dental workforce could help enhance oral health equity, notably through enhanced quality and satisfaction of patient care experiences [15, 22]. Racial and ethnic minorities have historically been underrepresented in dentistry in the United States [22]. Some studies in Canadian provinces and Australian states have shown considerably greater dentistry workforce concentrations in urban cores compared with more rural areas [23, 24]. At the same time, the proportion of women in dentistry – once a predominantly male profession – is gradually increasing, with largely unknown but potentially important impacts for locations and modes of practice [25]. Despite workforce feminization, a small but growing body of literature points to enduring gender-based earnings gaps among dentistry practitioners [26–28]. Devaluation of women’s labour risks exacerbating challenges for the health and dental care sector to attract and retain talent [28, 29].

Major deficiencies persist in the availability, quality, and use of HROH data to inform oral health service improvements and redress inequalities within the dental workforce, particularly allied dental personnel [25, 30–32]. The evidence gap is aggravated by the large proportions of dentists working in the private sector in most countries, hindering the representation, consistency, and comparability of traditional data sources such as surveys and administrative records of dental practices [20, 33]. Inadequate knowledge about the impacts of health workforce financing policy may have unintended consequences, such as exacerbating gender-inequitable provider outcomes [34]. Population-based sources, including national census sources, are commonly used to model demand for dental services [20], but have been underused for estimating health workforce supply and distribution. This national observational study leverages census data to assess imbalances in the dental workforce in Canada, a policy context of rapid scaling up of publicly-funded dental coverage on the path to universality. Following the framework conceptualized by Zurn et al. [21], we identify and quantify potential HROH imbalances by occupational, geographical, institutional, and gender-related factors. Special attention is paid to the interplay of gender, occupation, and earnings, with aim to build the (scant) evidence base on how public health financing policy may be contributing, positively or negatively, to gender equity in the dental workforce.

## Methods

### Study setting

As in many countries, issues of spatial, gender, and other imbalances in the dentistry and allied health professional workforces in Canada are understudied [35]. Under Canada’s multi-stakeholder health system governance model, public funding for basic dental services is organized at the level of the provinces and territories, yielding inconstant coverage across jurisdictions and sociodemographic groups [33]. Most Canadians access oral health care through private dental offices, with services paid for out-of-pocket and/or through voluntary private insurance [8, 36]. Dentists in private practice set their patient fees by type of procedure or service, although provincial and territorial dental associations suggest annually updated fee guides. The organization of the dentistry workforce thus differs starkly from the physician workforce, for which financing is ensured across the country through a single-payer universal medical coverage system. The education and licensing of dental practitioners are also regulated at the level of provinces and territories. Some reports point to a growing pool of registered dentists in large urban centres, while rural and remote communities remain underserved [37]. The dental hygiene, dental therapy, and dental assisting workforces are characterized with variations in training requirements and scopes of practice across jurisdictions and over time [37].

A new federally-funded public dental insurance plan, being phased in over the period 2022–2025, is intended to accelerate universal access to the treatment of oral disease, notably by complementing provincial and territorial dental plans and reducing the financial burden among patients lacking private insurance [8, 36, 38, 39]. While the federal plan recognizes the crucial role of HROH in service delivery [39], it does not explicitly address potential impacts of labour supply, mix, or distribution to fill existing coverage gaps. Like in other countries, concerns have been raised whether dental offices are prepared to anchor their practices in person-centredness and have enough hygienists and assistants to respond to increasing numbers of patients seeking professional oral healthcare services [38, 40].

### Study design

We sourced microdata from the latest 2021 Canadian population census, conducted by Statistics Canada. The census includes a long-form questionnaire, which captures detailed sociodemographic and labour market information from a 25% sample of the household population. The 2021 individual response rate to the long-form was 95.7% [41]. The target population for this study consisted of all respondents who self-reported their main occupation in selected dental care professions, in the core working ages of 25–54 years, having postsecondary educational attainment, and who earned professional income in the previous two years.

We analyzed a set of key indicators of occupational distribution, geographic distribution, service sector, and gender representation. We included three occupational groups aligned with the primary tasks performed in jobs, based on the systematic taxonomy of the 2021 National Occupational Classification: dentists; dental hygienists and therapists; and dental assistants and laboratory assistants (Table 1) [42].

**Table 1:** Selected dental care professions, National Occupational Classification (NOC) 2021

Occupation	NOC code	Description
Dentists	31110	Diagnose, treat, prevent, and control disorders of the teeth and mouth. They work in private practice or may be employed in clinics, hospitals, public health facilities, and educational institutions.
Dental hygienists and dental therapists	32111	Provide dental hygiene services related to oral disease and mouth injury prevention, and limited restorative dental treatment. They are employed in a variety of settings including dentists’ offices, clinics, hospitals, public health agencies, educational institutions, or they may be self-employed.
Dental assistants and dental laboratory assistants	33100	Assist dentists, dental technologists, dental hygienists, and dental therapists with the examination and treatment of patients, preparation of dentures and other dental devices, and clerical functions. They work in dentists’ offices, community health centres, clinics, dental laboratories, and educational institutions.

Source: Adapted from Employment and Social Development Canada

To facilitate geographic analyses across Canada’s vast physical landscape, we linked the census data deterministically by respondents’ place of residence to the Index of Remoteness (IR), a geocoded measure of accessibility and connectivity for all inhabited communities (5,161 census subdivisions) [43]. Developed by the national statistical agency, the continuous index is based on a spatial gravity model for gauging communities in terms of population size, proximity to population centres, and accessibility to services and transportation infrastructures [44]. The index is considered useful to help nuance heterogeneous population needs across the country’s rural and remote areas, given that the most urbanized and accessible areas represent only 6.1% of the total landmass [45]. To enhance meaningful comparisons, we ranked communities by IR deciles into four categories: highly urbanized and accessible areas (decile 1), accessible areas (decile 2), moderately accessible areas (decile 3), and more rural and remote areas (deciles 4–10).

Places of work for dental practitioners were delineated according to the North American Industry Classification System, specifically: (i) ambulatory healthcare services, such as dentist offices, diagnostic laboratories, and other outpatient facilities (NAICS code 621); or (ii) any other subsector (e.g., hospitals and other service settings) [46]. Other tracers of potential institutional imbalances included work status (full-time versus part-time) and worker class (self-employed versus employee).

Gender was based on self-identification as a woman or man (with a very small number of non‑binary persons being distributed in the other two gender categories) [47].

### Statistical analyses

Following common approaches for HROH planning applications [20], we started with descriptive analyses of dental workforce size and distribution, including calculating workforce-to-population densities by occupation and geographies. Second, we conducted bivariate analyses of the interplays between gender, occupation, and earnings. Person-level annual earnings data were captured in the census from integrated administrative income tax and benefits records, including all wages from paid employment and net self-employment income (before income taxes and deductions) in the preceding calendar year [47]. The earnings data were logged to address skewness.

Third, we assessed gender inclusion in the dental workforce by means of multivariate econometric decomposition of earnings differentials between women and men for each of the three occupational groups. The wage equations for the counterfactual decomposition technique, known as the Blinder-Oaxaca method [48, 49], are based on two single-gender regressions as follows:

1 $$ln\!\left({earnings}_{M}\right)=\:{X}_{M}{B}_{M}+{\epsilon}_{M}$$

2 $$ln\!\left({earnings}_{F}\right)=\:{X}_{F}{B}_{F}+{\epsilon}_{F}$$

where $\:{X}_{i}$ denotes predictors of differed wages for both males and females, with $\:\beta\:$ representing its estimated parameter, and $\:\epsilon\:$ indicating the associated error. Differences in mean (logged) earnings between men and women, in relation to the predicted mean earnings specific to each gender, distinguish between the “explained” moderators of wage differences (i.e., those attributable to the observed characteristics of men and women as included in the models) and to estimate any residual or “unexplained” component. A significant unexplained portion of the gender wage gap is often termed in the literature as the effect of discrimination and other (unmeasured and unmeasurable) structural problems in the labour market [27, 50]. In addition to adjusting for geographic and institutional factors, we controlled for various professional and personal characteristics including age group, advanced university qualifications, household status (whether living with a marital partner and/or children), ethnic/ancestral minority status (whether the person identified as Indigenous or a visible minority versus Caucasian or white), and adult migrant status (whether the person immigrated to Canada in adulthood, i.e., at ages 18 or over).

The de-identified census microdata used in the analyses were accessed in the secure computing facilities of the Statistics Canada Research Data Centre at the University of New Brunswick (Fredericton, Canada). Individual-level sampling weights were applied to ensure population representation of the descriptives and robust 95% confidence intervals (CIs) for results of the bivariate and multivariate analyses. Population counts were rounded and all statistical outputs were subject to risk-based confidentiality vetting in respect of Statistics Canada data privacy guidelines. The decomposition methods were implemented using the Stata v16 statistical software [51].

## Results

### Descriptives of the dental workforce

The census data tallied an active oral health workforce of 12,380 dentists, 20,885 dental hygienists and therapists, and 19,780 dental assistants aged 25–54. In other words, in terms of occupational mix, there were 1.7 dental hygienists/therapists and 1.6 dental assistants for every practicing dentist. The counts represented workforce densities of 3.4 dentists per 10,000 population, 5.7 dental hygienists and therapists per 10,000 population, and 5.4 dental assistants per 10,000 population.

**Fig. 1:**
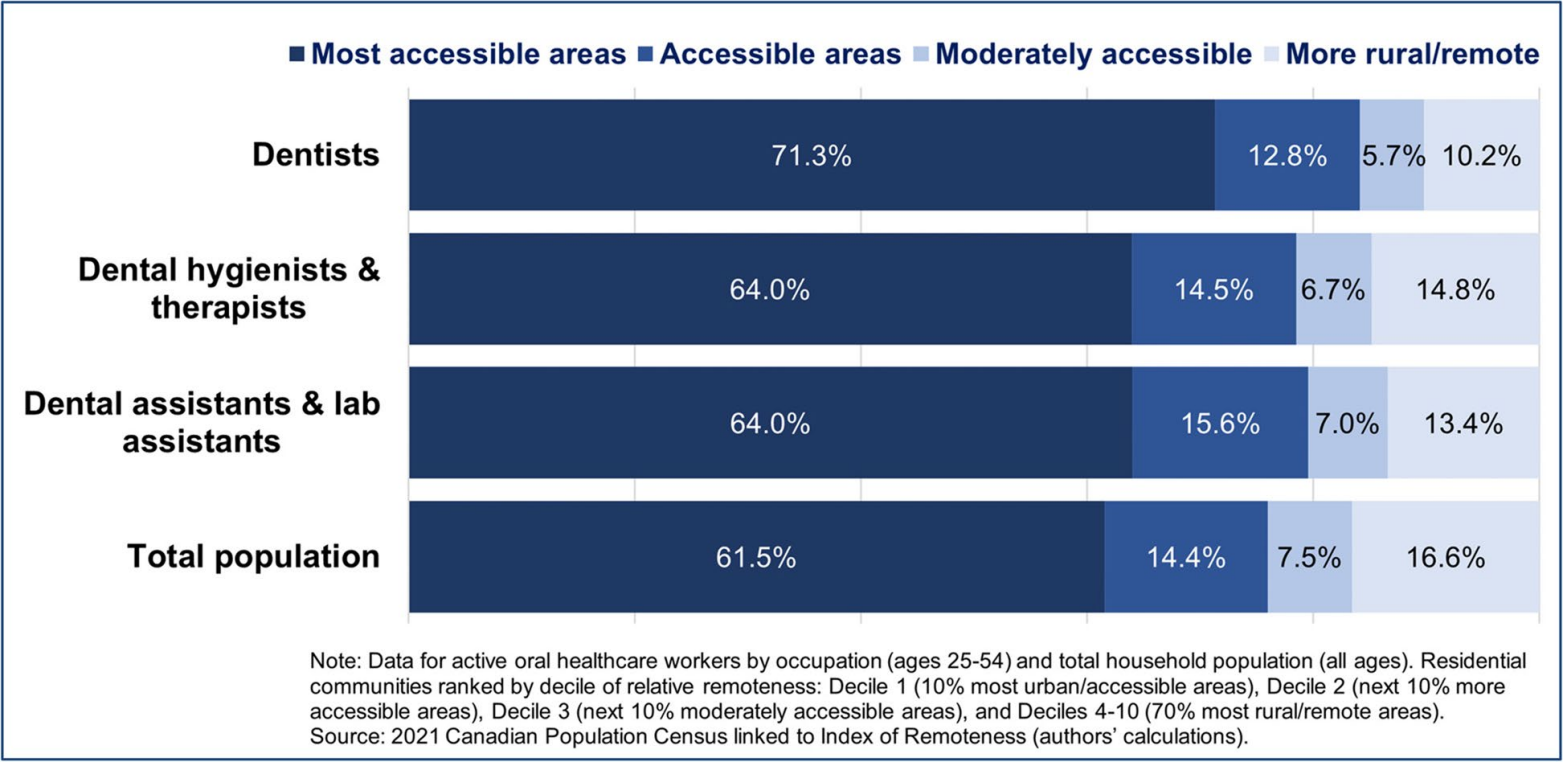
Geographic distribution of the dental workforce by relative remoteness of communities

Only 10% of dentists, 15% of dental hygienists, and 13% of dental assistants were located in more rural and remote areas of the country (IR deciles 4–10), despite 17% of the total population residing in these regions (Fig. 1). These geographic imbalances translated to workforce-to-population ratios in the most urbanized communities compared with more rural areas as being 1.9 times higher among dentists, 1.2 times higher among dental hygienists, and 1.3 times higher among dental assistants.

**Fig. 2:**
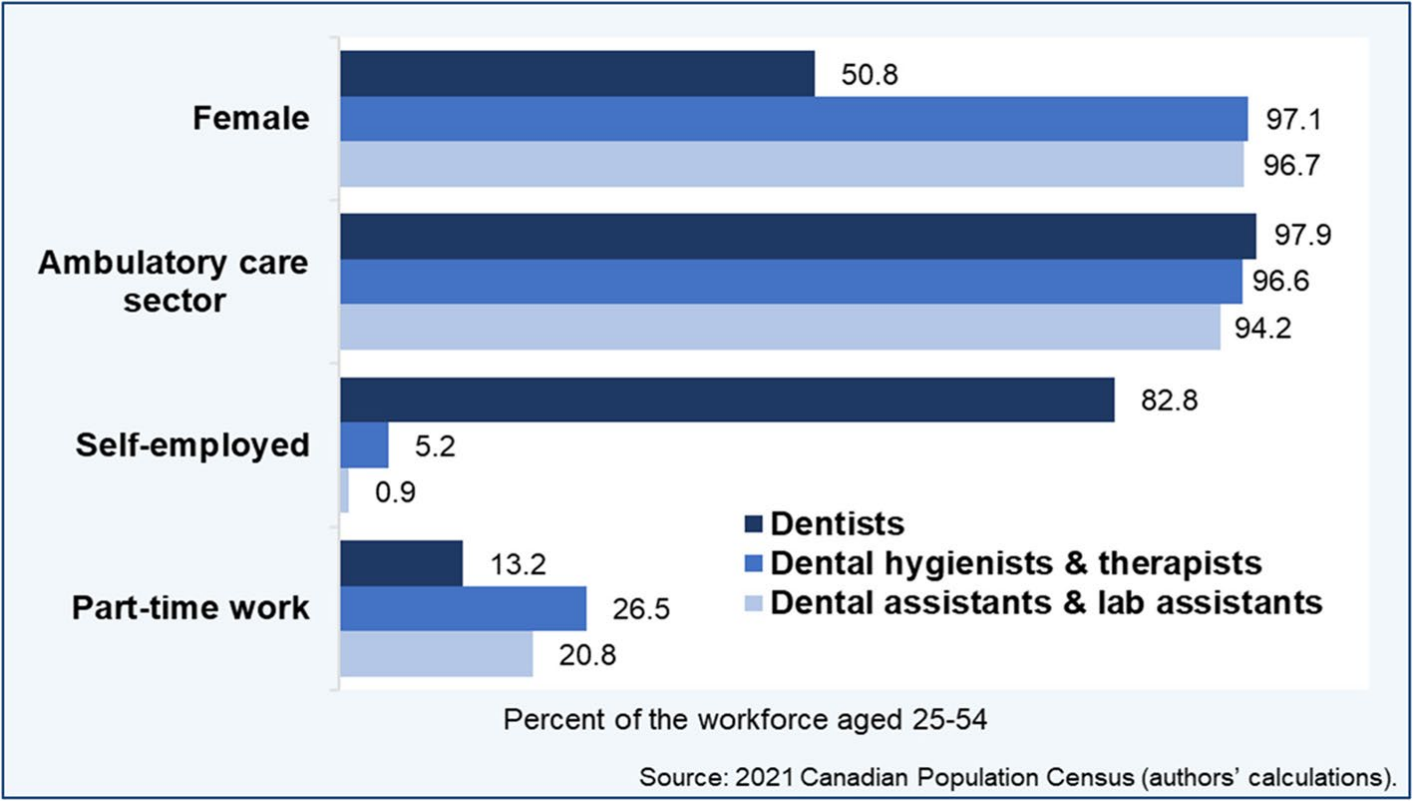
Institutional and gender distributions of the dental workforce

The dentistry profession was found to have achieved gender parity numerically (51% women), whereas both dental hygienists and dental assistants were predominantly female workforces (~ 97%) (Fig. 2). Regarding the institutional sector, a strong majority of HROH worked in ambulatory care services, and this across the three occupations (94–98%). Most dentists (83%) were self-employed, in contrast with few hygienists (5%) and even fewer assistants (< 1%). Fewer dentists worked mainly part-time (13%) than dental hygienists (27%) or assistants (21%). Not surprisingly, considerably more dentists had completed graduate-level university studies (17%) compared with allied service providers (1–2%) (not shown).

### Bivariate analysis

Disaggregating the geographic and institutional distributions of dental workers by gender, female dentists were underrepresented compared with their male counterparts in terms of rural practice: 9% versus 11% in the more rural and remote communities (Table 2). More female dental assistants than males were rural providers (14% versus 7%); no proportional difference was seen across female and male dental hygienists in rural areas (15% each). Female dentists were found equally as often as males to be working in ambulatory care services (98% each), but female dental assistants worked in ambulatory care much more often than males (95% versus 75%). On average, female dentists were found to earn 18% less annually than men. Among dental hygienists, the (unadjusted) gender earnings gap was 21%, whereas among dental assistants females tended to earn more than males.

**Table 2:** Indicators of potential geographic, institutional, and earnings imbalances in the dental workforce by gender

Characteristic	(1) Dentists	(2) Dental hygienists & therapists	(3) Dental assistants & lab assistants
**Geographic:** **Percent in more rural/remote areas**			
Men	11.2%	14.8%	7.0%
Women	9.3%	14.8%	13.6%
**Institutional**: **Percent outside of ambulatory care services**			
Men	2.1%	9.1%	25.4%
Women	2.1%	3.3%	5.1%
**Labour market inclusion**: **Mean annual professional earnings**			
Men	$ 122,835	$ 58,089	$ 36,751
Women	$ 93,526	$ 46,194	$ 33,563
**Gender earnings gap** (%, with 95% confidence interval)	-17.5%* (-12.9 – -21.9%)	-20.9%* (-14.9 – -26.5%)	15.1%* (4.6–26.7%)

Workforce data for wage earners aged 25–54 (earnings measured in 2020 Canadian dollars)

Source: 2021 Canadian Population Census linked to Index of Remoteness (authors’ calculations)

* = *p* < 0.05 (based on simple linear regressions predicting women’s mean log earnings in reference to men’s)

### Multivariate analysis: gender wage gaps in the dental workforce

The regression-based decomposition analyses revealed that female dentists earned 21% (95% CI: 11–32%) less than their male counterparts, after adjusting for geographic, institutional, and other professional and personal factors (Table 3, model 1). Much of the difference was explained statistically by differences between the female and male dentistry workforces in full-time versus part-time work status, age group, and immigrant status; however, part of the gap remained unexplained by the measured characteristics.

Although dental hygienists were 97% female, they earned on average 26% (95% CI: 13–42%) less than their male counterparts, all else being equal (Table 3, model 2). Part of the differential was explained by gender differences in work status, worker class, and personal characteristics (including presence of children in the home), but there remained a significant unexplained residual. Among dental assistants, any raw gender-based earnings difference was essentially explained by the measured predictors, notably observed differences between the female and male workforces in geographic, age group, and other characteristics (Table 3, model 3).

Working in the ambulatory care service sector and ethnic/ancestral minority status were not found to be independent predictors of gender earnings differences across each of the three professional groups.

**Table 3:** Explained and unexplained components from the Blinder-Oaxaca decompositions for female-male earnings differentials in the dental workforce

Predictor	(1) Dentists	(2) Dental hygienists & therapists	(3) Dental assistants & lab assistants
**Adjusted earnings differential ** (%, with 95% confidence interval)	-21.2%* (-11.3% – -32.0%)	-26.4%* (-12.7% – -41.8%)	13.2% (-3.3% – 26.9%)
**Explained**	59.0%*	1.2%	60.5%*
** Geographic:**			
Relative remoteness	1%	-179%	9%*
** Institutional:**			
Ambulatory care vs. other sectors	<1%	-99%	-25%
Full-time vs. part-time work	60%*	1869%*	-24%
Self-employed vs. employee	<1%	-1541%*	13%
** Other characteristics:**			
Age group	16%*	-726%	67%*
Advanced educational attainment	1%	304%	44%*
Household living arrangements	4%	1078%*	-37%*
Adult migrant status	20%*	-495%*	35%*
Ethnic/ancestral origin	-2%	-111%	18%
**Unexplained**	41.0%	98.8%*	39.5%

Workforce data for wage earners aged 25-54. Numbers in parentheses represent 95% confidence interval in the adjusted gender earnings gap (three models representing each professional group)

Source: 2021 Canadian Population Census linked to Index of Remoteness (authors’ calculations)

* = *p*<0.05

## Discussion

This population-based study quantified different domains of workforce imbalance salient to HROH planning on the path to universal dental coverage. While there is no “ideal” number or occupational mix of dental providers in national health systems [30], we found the dentists-to-population ratio in Canada to be nearly twice as high in the most urbanized and accessible regions compared with more rural and rural areas. Geographic imbalances were less pronounced among dental hygienists and dental assistants. The tendency for the dentistry workforce to remain concentrated in heavily urbanized regions echoed earlier findings in Canada [23, 35] and elsewhere around the world [30, 52]. Plans to reduce socioeconomic disparities in access to dental services among uninsured/underinsured Canadians by means of enhanced publicly-funded financial risk protection may thus be hampered in the absence of concerted efforts to address geographic imbalances in the dentistry workforce. The latter could entail, for example, operationalizing a “rural road map” for expanding dentistry education models to support rural workforce recruitment and retention, as currently existing for medical education [53]. In particular, it is noted that none of the dentistry schools in Canada are located in rural or small town communities [37]. There is little evidence that financial incentives alone contribute to longer-term rural retention of dentists and other allied health professionals [35, 54].

Important gender imbalances in the dental workforce were also exposed. While the dentistry profession had reached numerical gender parity among those aged 25–54, a significant gender-based earnings gap persisted, with female dentists earning some 20% less than their male counterparts after adjusting for geographic, institutional, and other professional and personal factors. Such findings were consistent with a study on trends in the gender wage gap among the dentistry workforce in the United States, which reported that – despite a convergence between women and men in observable characteristics over time (e.g., age, work status, race, children) – an unexplained residual surfaced from the decomposition analysis [27]. We further found that male dental hygienists earned significantly more than females, despite men representing merely 3% of this workforce. It is possible that a “glass escalator” may be at play here, i.e., a metaphor for the observed pattern of men in women-dominated occupations being more likely to receive promotions and other workplace rewards compared with their women peers [29]. The glass escalator has been theorized to explain higher wages among men in other healthcare occupations heavily dominated by women, e.g. among nurses and nursing assistants [29]. It has been speculated elsewhere that men and women dental care workers practice differently, such as women dentists being more likely to favour preventive approaches over surgical specialties, although more robust data are needed [25]. Further research on gender imbalances and discrimination is required to disentangle gender-specific practice differences and provider outcomes and help inform gender-responsive HROH financing policy [20, 34]. For example, a survey of dentists in private practice in the United States found women tended to work slightly fewer hours than men, but were more likely to provide care for patients covered by public dental insurance [55]. In Canada, early anecdotal evidence suggests the numbers of dentists enrolled in the new federal government insurance plan intended to achieve universal dental coverage have been insufficient to meet growing patient demand, partly due to concerns over lower compensation rates under the federal plan than those recommended by provincial/territorial dental associations [56]. Oral healthcare financing arrangements that do not address underlying imbalances in the dental workforce may thus miss significant opportunities for gender equity promotion.

In leveraging population census data, this study highlights the need for greater use and re-use of official statistical sources for HROH analyses. A small but growing body of literature is drawing on census data for allied health workforce research (e.g., [24, 35]). Compared with studies relying on health professional registries, the census allows for collating nationally-representative data among active mid-level providers and support staff regardless of license issues — which can vary across jurisdictions and over time. For example in Canada, dental hygienists are not regulated consistently across provinces and territories [37]. While there is no unanimous definition of who comprises the oral health workforce, data-driven research and planning often focus only on dentists, who mostly require licensing for professional practice around the world [32].

As with all observational studies, certain limitations are acknowledged. One lies in the absence of available comparable data on practice and patient profiles for estimating essential oral healthcare needs, beyond basic provider-to-population ratios. Increasing patient demand for private dental services is being driven in part by rising popularity of new cosmetic dental techniques, that is, for largely aesthetic reasons [31]. While we measured rural heterogeneity through a novel index of remoteness and accessibility for all inhabited communities, privacy protocols governing the census data precluded us from disaggregating labour market imbalances for some specific occupations (e.g., denturists and other numerically smaller cadres) or from mapping precise geographic locations of dental practitioners. For example, a previous analysis of 2016 Canadian census data grouping multiple allied health professions indicated that the provider-to-population fell steeply in the 10% most remote communities (i.e., IR decile 10) [35]. Lastly, while the 2021 census included newly expanding questions on gender identity, only a binary gender variable (woman or man) was available for research use at the time of this study.

## Conclusions

This Canadian study on HROH enumerated a density of 3.4 active dentists aged 25–54 per 10,000 population, supported by an allied workforce of 1.7 dental hygienists/therapists and 1.6 dental assistants for every dentist. Almost all dental providers were working in ambulatory care services (94–98%), although the proportion was lower among male dental assistants (75%). In a national context of universal coverage for medical (but not dentistry) services, the oral health workforce was demarcated by considerable geographic and gender-based imbalances. The density of dentists was nearly twice as high in the most urbanized regions compared with rural and rural areas, and female dentists earned 21% less on average than their male counterparts. Despite women representing 97% of dental hygienists/therapists, they earned 26% less on average than males. More cross-nationally comparable research is needed to inform and benchmark innovative approaches for equity-oriented HROH planning, often critically overlooked in public policy for health systems strengthening.

## Data Availability

The census data that support the findings of the study are available through Statistics Canada’s Research Data Centres but restrictions apply to the availability of these confidential data, which were used with permission for the current study, and so are not publicly available.

## Abbreviations

CI: Confidence interval
HROH: Human resources for oral health
IR: Index of Remoteness
NAICS: North American Industry Classification System
NOC: National Occupational Classification
SDGs: Sustainable Development Goals
